# Antibiotics susceptibility patterns of uropathogenic bacteria: a cross-sectional analytic study at Kanifing General Hospital, The Gambia

**DOI:** 10.1186/s12879-023-08373-y

**Published:** 2023-10-25

**Authors:** Abou Kebbeh, Paul Dsane-Aidoo, Kawsu Sanyang, Sheriffo M. K. Darboe, Nuha Fofana, Donne Ameme, Abdoulie M. Sanyang, Kalifa Sanneh Darboe, Saffiatou Darboe, Bakary Sanneh, Ernest Kenu, Francis Anto

**Affiliations:** 1 Ghana Field Epidemiology and Laboratory Training Program, Department of Epidemiology and Disease Control, School of Public Health, University of Ghana, Accra, Ghana; 2 National Public Health Laboratories, Ministry of Health, Banjul, The Gambia; 3 World Health Organization, Accra, Ghana; 4 Kanifing General Hospital, Ministry of Health, Banjul, Gambia; 5 Laboratory Management, Medical Research Council Unit at the LSTHM, Banjul, The Gambia

**Keywords:** Urinary Tract Infections, Uropathogenic bacteria, Antibiotics, Susceptibility, Antimicrobial resistance, The Gambia

## Abstract

**Background:**

Antimicrobial resistance poses a public health threat for the treatment of community-acquired urinary tract infections. This study determined the susceptibility patterns of uropathogens and associated risk factors among outpatients diagnosed with urinary tract infections at the Kanifing General Hospital in the Gambia.

**Methods:**

A cross-sectional analytic study was conducted among patients with suspected urinary tract infections at Kanifing General Hospital from March to May 2021. Data on socio-demographic and other risk factors were collected from the study participants using a structured pre-tested questionnaire. Mid-stream urine samples were collected, and bacteria identification and antimicrobial susceptibility testing done using standard microbiological methods. Descriptive and inferential statistical analysis were done to determine factors associated with urinary tract infection at 95% confidence level and a *p* -value < 0.05.

**Results:**

A total of 422 patients were enrolled with 82.5% (348/422) being females. The prevalence of community acquired urinary tract infection was 12.8% (54/422). *Escherichia coli* was the most prevalent isolate (74.1%, 40/54), followed by *Klebsiella* spp (8.5%, 10/54). Antimicrobial resistance was highest for Ampicillin (87.0%, 47/54), Trimethoprim/Sulfamethoxazole (77.8%, 42/54) and Tetracycline (75.9%, 41/54). Uropathogens sensitivity was 77.8% (42/54) for Nitrofurantoin and 75.9% (41/54) for Ceftazidime. Being female (aOR 5.90 95% CI = 1.48–23.67), previous history of urinary tract infection (aOR 2.34, 95% CI = 1.06–5.14), use of unprescribed antibiotics (aOR 2.0, 95% CI = 1.05–3.62) and having no formal education (aOR 8.02, 95% CI = 1.04–62.0) were significant factors associated for having uropathogenic bacterial infection.

**Conclusion:**

*E. coli* was the most prevalent uropathogen isolated. Ciprofloxacin, Nitrofurantoin and Ceftazidime were the most sensitive antibiotics. Routine surveillance of susceptibility of uropathogenic bacteria would be helpful to update clinicians on the choice of antibiotics.

## Background

Urinary Tract Infections (UTIs) are among the most frequent community- and hospital-acquired infections with an increasing antibiotic resistance [1]. The disease is predominantly caused by Gram-negative bacteria including *Escherichia coli*, *Klebsiella* spp, *Proteus mirabilis, Pseudomonas aeruginosa* and Gram-positive bacteria such as *Staphylococcus saprophyticus* [2]. Globally, about 150 million people are diagnosed with UTIs yearly, resulting in an estimated health care expenditure in excess of six billion dollars [3].

The prevalence of uropathogens is associated with several factors including age, poor economic status, poor hygiene, hospitalization, catheterization, sexual activities, pregnancy and diabetes [4–7]. The infections are common in young, sexually active women, with incidence exceeding 0.5 episodes per person per year, and about 30% experiencing recurrent infections [8]. Reports from Sub-Saharan Africa show varying prevalence levels among different populations and age-groups; 89.17% among female patients in Nigeria [1], 39.13% among adult outpatients in Uganda [9], 10.1% in Ghana [10], 26.7% among adult patients in Senegal [11] and 21.2% among Gambian children [12].

Antimicrobial misuse in the management of UTIs contributes significantly to the development of antimicrobial resistance (AMR). AMR was estimated to cause about 700,000 deaths per year globally, and if the present trend continues, it might cause over 10 million deaths per year by 2050. In Africa, if action is not taken an estimated 4.1 million people could die as a result of treatment failure by 2050 [13]. This projection has recently been enforced with Western sub-Saharan Africa having the highest burden with 27.3 deaths per 100,000 attributable to AMR and 114.8 deaths per 100,000 associated with AMR in 2019 [14]. High levels of AMR have been reported in West Africa -76.8% in Nigeria [15], including high levels of multidrug resistance (MDR); 80.1% in Ghana [10], 60% in Senegal [11] and 9.0% in The Gambia [16].

In The Gambia, very few reports on the antibiotic susceptibility patterns of uropathogenic bacteria are available [16]. Most health facilities rely on urinalysis and urine microscopy results for treatment without performing antimicrobial susceptibility tests. As a result, clinicians frequently use empirical therapy without knowing the sensitivity of the specific antibiotics being used. With the emergence of antibiotic resistance in the Gambia [12, 17, 18], clinicians need information on locally prevalent uropathogenic bacteria strains and their susceptibility patterns to ensure antimicrobial stewardship. This study determined the antibiotics susceptibility patterns and factors associated with uropathogenic bacterial infection in patients with Community Acquired-Urinary Tract Infections (CA-UTIs) attending the Kanifing General Hospital (KGH) in The Gambia.

## Materials and methods

### Study design

A cross-sectional analytical study was conducted among clients attending the Out-Patients-Department (OPD) of the Kanifing General Hospital. Patients clinically suspected of community-acquired urinary tract infection (CA-UTI) by attending clinicians at the OPD and referred for laboratory confirmation from March to May 2021 were consecutively recruited. Mid-stream urine samples were received from the patients and culture and sensitivity test performed. Data on demographic characteristics, risk factors for urinary tract infection, including previous UTI, catheterization, and antibiotic use were collected from the patients using a case report form and a structured questionnaire.

### Study setting

The Kanifing General Hospital (KGH) is located in Kanifing Municipal Area Council (KMC), which is one of the eight Local Government Areas (LGAs) in The Gambia [19]. The municipality is one of the most densely populated areas and lies in the western part of Banjul, the capital city. The municipality includes Serekunda, which is the largest urban area in the Gambia. According to the Gambia Bureau of Statistics, the population of Kanifing LGA is 382,096 [19]. The hospital is one of the main tertiary care centers in the country that provides health care services to a large section of the population in KMC. The hospital receives patients mainly from the urban area and is the second major point of referral within the urban area. The hospital has a total bed capacity of 340. In addition to general medical services, the hospital provides surgical, laboratory and radiography services.

### Study population and sampling techniques

A total of 422 urine samples were estimated for this study using the Cochran formula [20]. Patients who were clinically diagnosed with CA-UTI at the OPD by clinicians and referred to the laboratory for confirmation were consecutively enrolled into the study until the predetermined sample size was obtained. Patients already on antibiotic treatment for any condition and pregnant women mothers in labour were excluded from the study [21].

### Data and specimen collection

Data on socio-demographic characteristics (sex, age, employment, education and marital status), risk factors for UTI (history of UTI, pregnancy, urethral catheterization, hospitalization, history of diabetes mellitus), were collected directly from participants using a structured questionnaire and case report forms. Also, information on antibiotic usage (consumption within the last three months, purchase from street vendors, self-medication, adherence to prescription and sharing of left-over antibiotics) were collected.

After detailed instructions, study participants were given a clean, sterile screw-capped labeled container to collect clean-catch midstream urine. A volume of 5–10 ml of urine specimen was requested from each patient. For female patients, they were informed to do handwashing and cleanse the area around the urethral opening with clean water, dry with sterile gauze and collect the urine with the labia held apart. Male patients were instructed to wash hands prior to collection and collect urine in the middle of the urine flow [22]. The collected samples were analyzed within one hour after collection [23]. Children who were too young to follow aseptic instructions, were supported by parents.

### Sample processing and identification of isolates

Using calibrated inoculating loop 0.001 ml, each urine sample was inoculated on Cysteine Lactose Electrolytes Deficient (CLED) agar plates and incubated at 37 °C for 18–24 h. After overnight incubation, colonies were counted for the estimation of bacterial load per milliliter of urine sample [24]. A specimen was considered positive for UTI if the isolates cultivated had a concentration of ≥ 10^5^ cfu/ml. After 24 h, the bacterial colonies with pure and significant growth were further confirmed using standard biochemical tests. All positive urine cultures with significant bacteriuria were identified using Analytical Profile Index 20E (API 20E) (bioMerieux) identification system [25].

### Antimicrobial susceptibility testing

Antimicrobial Susceptibility Testing (AST) was done using Kirby Bauer’s disc diffusion method following standard guidelines of Clinical and Laboratory Standard Institute (CLSI) [26]. The isolates were tested for their resistance using the following antibiotics that are locally available; Ampicillin (AMP 10 µg), Cefotaxime (CTX 30 µg), Amoxicillin-clavulanic acid, Gentamycin (GEN) (10 µg), Tetracycline (TET 15 µg), Chloramphenicol (CHL 10 µg), Nalidixic acid (NAL 30 µg), Ciprofloxacin (CIP 5 µg), sulfamethoxazole/trimethoprim (SXT 25 µg) and Imipenem (I 10 µg) [17]. ESBL phenotypic was determined based on a ≥ 5 mm increase in a zone diameter for either Cefotaxime or Ceftazidime antibiotics tested in combination with Clavulanate versus the zone of inhibition of the antibiotics when tested alone [26]. Multidrug resistance was defined as bacteria being resistant to three or more antimicrobial classes [27].

### Quality control

The laboratory analysis was conducted using Standard Operating Procedures. The culture media were tested for both sterility and performance by inoculating 5% of the batch after preparation [28]. Standard reference strains of *Escherichia coli* ATCC 25922, *Klebsiella pneumoniae* ATCC 700603 were used during culture and AST to ensure accuracy of tests done.

### Data analysis

Data were entered into Microsoft Excel, cleaned, coded, and analyzed using Stata software version 16.1. All statistical tests were performed at a 95% confidence level. Prevalence in UTI and antibiotic susceptibility patterns were described using proportions. Binary logistic regression analysis was carried out, and odds ratios (OR) [crude odds (cOR) and adjusted odds (aOR)] estimated to determine association between each of the independent variables and dependent variable (uropathogenic bacterial infection). Variables with *p* < 0.05 at 95% confidence level were considered statistically significant.

## Results

### Background characteristics of study participants

A total of 422 patients participated in the study, most of them female (82.5%, 348/422), married 67.5% (285/422), median age, 30 years (IQR = 30—40 years), and 46.2% (195/422) without formal education (Table 1).

**Table 1:** Demographic characteristics of the study participants visiting Kanifing General Hospital, 2021

Characteristics	Frequency	Percentage
**Sex**		
Male	74	17.5
Female	348	82.5
**Marital status**		
Single	123	29.2
Married	285	67.5
Divorce	14	3.3
**Pregnancy Status**		
Pregnant	67	19.3
Not pregnant	281	80.8
**Level of Education**		
No formal education	195	46.2
Primary	40	9.5
Secondary	123	29.2
Tertiary	64	15.2
**Occupation**		
Not employed	223	52.8
Self-employed	83	19.7
Civil servant	46	10.9
Student	70	16.6

### Prevalence of uropathogenic bacteria infection

**Fig. 1:**
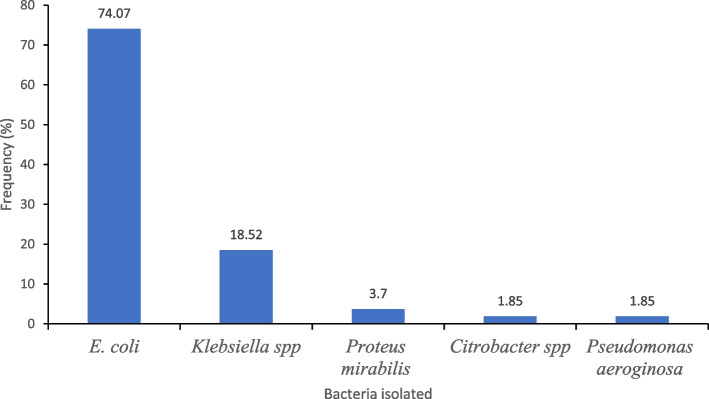
Isolates of uropathogenic bacteria isolated from study participants diagnosed with UTI, KGH, 2021

**Fig. 2:**
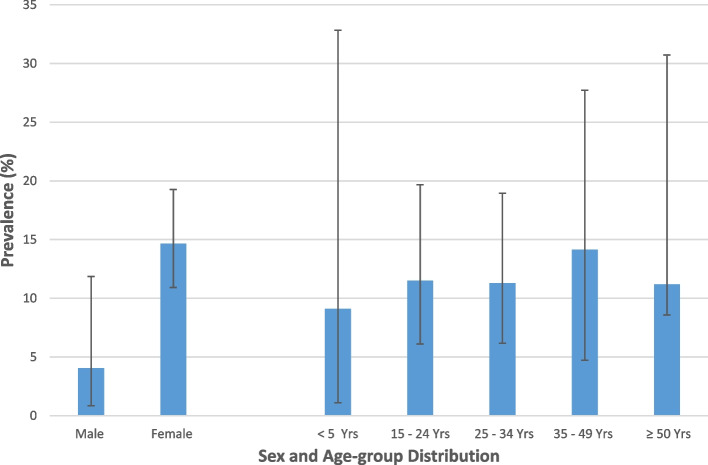
Prevalence of uropathogenic bacteria by sex and age-group. The points plotted (-) indicate the percentage of the age-group or sex infected, while the vertical lines show the corresponding 95% confidence intervals

A total of 54 samples yielded significant growth of uropathogenic bacteria (≥ 10^5^ cfu/mL); giving an overall prevalence of uropathogenic bacterial infection of 12.8% (54/422), 95% CI [0.098–0.164]. Bacteria belonging to five different Genera were isolated; these were, *Escherichia coli, Klebsiella* spp. *Pseudomonas aeruginosa, Proteus* spp. and *Citrobacter* spp. (Fig. 1). The predominant bacterial isolates were *E. coli* (74.1%, 40/54), and *Klebsiella* spp. (18.5% (10/54). A higher proportion of females 14.66% (51/348) than males 4.05% (3/74) had uropathogenic bacterial infection. The difference was statistically significant (*p* = 0.03). No significant difference was found in the prevalence of infection among the different age-groups (Fig. 2).

### Antibiotics susceptibility patterns of uropathogenic bacterial isolates

Among the isolates tested against various antibiotics, 87.0% (47/54) were resistant to Ampicillin. High proportions of the bacteria were also resistant to Tetracycline and Trimethoprim/Sulfamethoxazole (75.9%, 41/54) and (77.8%, 42/54) respectively. In addition, 61.1% (33/54) and 53.9% (29/54) of the isolates were also observed to be resistant to Ciprofloxacin and Nalidixic acid, respectively. However, Nitrofurantoin (77.8%, 42/54), Ceftazidime (75.9%, 41/54), Cefotaxime (72.2%, 39/54), Gentamycin (72.2%, 39/54) and Amoxicillin-clavulanic acid (68.5%, 33/54) were observed to be sensitive against the isolates. All the isolates were 100% sensitive to Imipenem (Table 2).

**Table 2:** Antimicrobial susceptibility pattern of uropathogenic bacteria isolated from participants with CA-UTI, Kanifing General Hospital, 2021 (*N* = 54)

Uropathogens		Antibiotics n(%)										
		AMC	CTX	AMP	NIT	GEN	CIP	TET	CHL	SXT	NA	I
***E. coli (*** **40)**	S	5(12.5)	31(77.5)	3(7.5)	34(85.0)	31(77.5)	25(62.5)	7(17.5)	32(80.0)	8(20.0)	18(45.0)	40(100)
	I	6(15.0)	1(2.5)	1(2.5)	1(2.5)	0(0.0)	0(0.0)	2(5.0)	3(7.5)	0(0.0)	1(2.5)	0(0.0)
	R	29(72.5)	8(20.0)	36(90.0)	5(12.5)	9(22.5)	15(37.5)	31(77.5)	5(12.5)	32(80.0)	21(52.5)	0(0.0)
***Klebsiella*** ** spp (10)**	S	5(50.0)	4(40.0)	NA	5(50.0)	5(50.0)	5(50.0)	2(20.0)	7(70.0)	3(30.0)	6(60.0)	10(100)
	I	3(30.0)	0(0)	NA	0(0.0)	0(0.0)	0(0.0)	0(0.0)	1(10.0)	0(0.0)	0(0.0)	0(0.0)
	R	2(20.0)	6(60.0)	NA	5(50.0)	5(50.0)	5(50.0)	8(80.0)	2(20.0)	7(70.0)	4(40.0)	0(0.0)
***Proteus mirabilis*** ** (2)**	S	1(50.0)	2(100)	1(50.0)	1(50.0)	1(50.0)	1(50.0)	1(50.0)	1(50.0)	1(50.0)	1(50.0)	2(100)
	I	1(50.0)	0(0.0)	0(0.0)	0(0.0)	0(0.0)	0(0.0)	0(0.0)	0(0.0)	0(0.0)	0(0.0)	0(0.0)
	R	0(0.0)	0(0.0)	1(50.0)	1(50.0)	5(50.0)	1(50.0)	1(50.0)	1(50.0)	1(50.0)	1(50.0)	0(0.0)
***Citrobacter*** ** spp (1)**	S	1(100)	1(100)	0(0.0)	1(100)	1(100)	1(100)	0(0.0)	1(100)	0(0.0)	0(0.0)	1(100)
	I	0(0)	0(0.0)	0(0.0)	0(0.0)	0(0.0)	0(0.0)	1(100)	0(0.0)	0(0.0)	0(0.0)	0(0.0)
	R	0(0)	0(0.0)	1(100)	0(0.0)	0(0.0)	0(0.0)	0(0.0)	0(0.0)	1(100)	1(100)	0(0.0)
***Pseudomonas aeruginosa*** ** (1)**	S	1(100)	1(100.0)	NA	1(100)	1(100)	1(100)	NA	NA	NA	NA	1(100)
	I	0(0)	0(0.0)	NA	0(0.0)	0(0.0)	0(0.0)	NA	NA	NA	NA	0(0.0)
	R	0(0)	0(0.0)	NA	0(0.0)	0(0.0)	0(0.0)	NA	NA	NA	NA	0(0.0)
**Total (** ***N*** ** = 54)**	S	37(68.5)	39(72.2)	4(7.41)	42(77.8)	39(72.2)	33(61.1)	11(20.4)	42(77.8)	12(22.2)	23(42.6)	54(100)
	I	8(14.8)	1(1.85)	3(5.6)	1(1.85)	0(0.0)	0(0.0)	2(3.7)	4(4.0)	0(0.0)	29(53.7)	0(0.0)
	R	9(16.67)	14(25.9)	47(87.04)	11(20.4)	15(27.78)	21(38.9)	41(75.9)	8(14.81)	42(77.8)	2(3.7)	0(0.0)

*S* Susceptible, *I* Intermediate, *R* Resistance, *AMC* Amoxicillin-clavulanic acid, *CTX* Cefotaxime, *AMP* Ampicillin, *NIT* Nitrofurantoin, *GEN* Gentamycin, *CIP* Ciprofloxacin, *TET* Tetracycline, *CHL* Chloramphenicol, *SXT* Trimethoprim/Sulfamethoxazole, *NA* Nalidixic acid, *I* Imipenem, *NA* Not Applicable

### Multi-resistance rates of uropathogenic bacteria by isolates.

Most of the isolates, 88.9% (48/54) were resistant to at least one of the 11 antimicrobial agents tested. Of this, 1.9% (1/54) showed resistance primarily to one antibiotic only, 11.1% (6/54) were resistant to two antibiotics, 13.0% (7/54) were resistant to three antibiotics, 16.7% (9/54) were resistant to four antibiotics and 57.4% (31/54) were resistant to five or more antibiotics. Overall, 87.0% (47/54) of the isolates were multidrug resistant (MDR) as they were resistant to three or more antibiotic classes. The highest frequency of MDR was observed in *Klebsiella* spp. occurring in nine samples and *E. coli* (35 isolates). No MDR was observed in *Pseudomonas aeruginosa* (Table 3).

**Table 3:** Distribution of multi-resistance uropathogenic bacteria recovered from patients by isolates type, Kanifing General Hospital, 2021

Isolates (N)	Degree of Resistance n(%)					Total MDR (≥ R3)	ESBL Producer (%)
	**R1**	**R2**	**R3**	**R4**	** ≥ R5**		
***E. coli*** ** (40)**	1(2.5)	4(10.0)	5(12.5)	7(17.5)	23(57.5)	35(87.5)	8(20)
***Klebsiella*** ** spp (10)**	0(0)	1(10.0)	1(10.0)	2(20.0)	6(60.0)	9(90.0)	5(50)
***Proteus mirabilis*** ** (2)**	0(0)	0(0)	1(50.0)	0(0)	1(50.0)	2(100)	0
***Citrobacter*** ** spp (1)**	0(0)	0(0)	0(0)	0(0)	1(100)	1(100)	0
***Pseudomonas aeruginosa*** ** (1)**	0(0)	1(100)	0(0)	0(0)	0(0)	0(0)	0
**Total (54)**	1(1.9)	6(11.1)	7(13.0)	9(16.67)	31(57.4)	47(87.0)	13(24.1)

R1-5: Resistance to 1, 2, 3, 4 and 5 antibiotics, ≥ R3 resistance to 3 or more antibiotics

### Phenotypic Extended-Spectrum Beta-Lactamase (ESBL) producing Organisms

Among the isolates, 24.1% (13/54) were positive for ESBL production with 50% (5/10) and 20% (8/40) isolates being *Klebsiella* spp and *E. coli* respectively (Table 3).

### Factors associated with uropathogenic bacterial infection

The multivariable analysis revealed that being female (aOR = 5.90(1.48–23.67), having no formal education (OR = 8.02(1.04–62.0)), having a previous history of UTI (aOR = 2.34(1.06–5.14)) and purchasing antibiotics from street vendors (aOR = 2.0(1.05–3.62)) were significantly associated with uropathogenic bacterial infection. However, history of urethral catheterization (aOR = 2.62, 95% CI = 0.90–7.64), having diabetes (OR = 2.43, 95% CI = 0.96–6.09) and taking antibiotics within the last three months (OR = 0.74 95% CI = 0.39–1.41) were not significantly associated with uropathogenic bacterial infection (Tables 4 and 5).

**Table 4:** Factors associated with urinary ttract infections among respondents, Kanifeng General Hospital

Variables	Uropathogenic Bacteria		cOR (95% CI)	*p*-value	aOR (95% CI)	*p*-value
	Negative n (%)	Positive n (%)				
**Sex**						
Male	71(96.0)	3(4.0)	1		1	
Female	297(85.3)	51(14.7)	4.1(1.23–13.30)	0.021	5.90(1.48–23.67)	0.021
**Occupation**						
Not employed	183(82.1)	40(17.9)	3.1(0.93–10.61)	0.066	NA	
Self-employed	77(92.8)	6(7.2)	1.11(0.26–4.70)	0.88	NA	
Student	65(92.9)	5(7.1)	1.10 (0.25–4.85)	0.897	NA	
Civil servant	43(93.5)	3(6.5)	1		NA	
**Level of education**						
No formal education	164(84.1)	31(15.9)	11.91(1.59–89.09)	0.016	8.02(1.04–62.0)	0.046
Primary	37(92.5)	3(7.5)	5.10 (0.51–50.91)]	0.164	5.05(0.48–52.45)	0.174
Secondary	104(84.5)	19(15.5)	11.50 (1.50–88.08)	0.019	9.28(1.18–72.61)	0.034
Tertiary	63(98.4)	1(1.6)	1		1	
**Marital status**						
Married	241(84.6)	44(15.4)	2.06(1.00–4.24)	0.049	NA	
Single	113(91.9)	10(8.1)	omitted		NA	
Divorce	14(100)	0	1		NA	
**Medical illness (Diabetes)**						
Diabetic	59(84.3)	11(15.7)	1.34 (0.65–2.74)	0.425		
Non-Diabetic	309(87.8)	43(12.2)	1		NA	
**Prior Hospital stay (last month)**						
Hospitalized	36(83.7)	7(16.3)	1.37 (0.58–3.26)	0.472	NA	
Not hospitalized	332(87.6)	47(12.4)	1		NA	
**History of catheterization**						
Catheterized	19(76.0)	6(24.0)	2.29(0.87–6.03)	0.092	NA	
Not catheterized	349(87.9)	48(12.1)	1		NA	
**History of UTI**						
Ever had UTI	241(84.9)	43(15.1)	2.05(1.03–4.13)	0.042	2.34(1.06–5.14)	0.033
No history of UTI	127(92.0)	11(8.0)	1		1

**Table 5:** Antibiotic usage factors associated with uropathogenic bacteria resistant isolates, Kanifing General Hospital

Variable	Uropathogenic Bacteria		cOR (95% CI)	*p*-value	aOR (95% CI)	*p*-value
	Negative n (%)	Positive n (%)				
**Antibiotic consumption during illness**						
Taken antibiotics	209(85.3)	36(14.7)	1.52 (0.83–2.78)	0.172	NA	
Not taken	159(89.8)	18(10.2)	1			
**Prior (3 months) antibiotic exposure without medical prescription**						
Without prescription	125(89.3)	15(10.7)	0.74 (0.39–1.41)	0.368	NA	
With prescription	243(86.2)	39(13.8)	1			
**Bought and used antibiotics from local pharmacy/street vendors**						
Bought and used	133(82.6)	28(17.4)	1.90(1.07–3.38]	0.028	2.0(1.05–3.62)	0.033
Did not	235(90.0)	26(10.0)	1			
**Usually take antibiotic without medical prescription**						
Usually take	114(83.8)	22(16.2)	1.53 (0.85–2.75)	0.154	NA	
Do not take	254(88.8)	32(11.2)	1			
**Finished dosage prescribed antibiotics**						
Finished dosage	210(86.1)	34(13.9)	1.27 (0.71–2.30)	0.413	NA	
Did not finish	158(88.8)	20(11.2)	1			
**Share antibiotic with family members or friends**						
Do share	64(81.0)	15(19.0)	1.83 (0.95–3.51)	0.064	NA	
Do not share	304(88.6)	39(11.27)	1			

*aOR* Adjusted odds ratio, cOR Crude odds ratio, *CI* Confidence Interval, *NA* Not applicable

## Discussion

This study determined the antibiotic susceptibility patterns of uropathogenic bacterial isolates and associated factors among outpatients attending the Kanifing General Hospital, in The Gambia. Of five Genera of bacteria isolated from urine samples of the study participants, the most predominant were *E. coli* (74.1%) and *Klebsiella* spp. (18.5%). Community-acquired UTIs were found to be associated with the level of formal education, history of urinary tract infection (UTI) and use of unprescribed antibiotics. The bacterial isolates were found to have some level of resistance to all the antibiotics studied except for Imipenem.

The overall prevalence of uropathogenic bacterial infection was 12.85%, which is not very different from that reported from some other African countries, including the Gambia (9.0%), Ghana (10%) and Madagascar (12.9%) [10, 16, 29]. This is however, much lower than the levels reported in Senegal (26.7%) and Ethiopia (21.1%)[11, 28]. The relatively low prevalence our study recorded could be due to the differences in sample size, duration of study period and the methods used in the identification of bacterial uropathogens. Prior antibiotic use is known to affect urine culture results as the medication inhibit bacterial growth [28, 30].

The antibiotics found to be most sensitive were the carbapenems (imipenem and meropenem) and cephalosporin (cefotaxime). It was observed that Nitrofurantoin, which is among the recommended antibiotics for the treatment of uncomplicated UTI in The Gambia demonstrated only moderate sensitivity. Ciprofloxacin and other fluoroquinolones are also among the recommended and broadly prescribed antibiotics for UTIs [3]. This current study found the bacterial isolates to be moderately susceptible to ciprofloxacin and Nalidixic acid. Therefore, these antibiotics may be reserved and only used after confirmation of sensitivity to specific uropathogens. These findings are consistent with earlier reports from Ethiopia [30], but in contrast with a study from Madagascar [29]. These antibiotics are among the most commonly used antibiotics in the Gambia since they are cheap and easy acquired from drug stores.

The bacterial isolates were found to be highly resistant to ampicillin, tetracycline, and trimethoprim/sulfamethoxazole, with eight out of ten of the isolates showing resistance to ampicillin. The high level of resistance renders these antibiotics unsuitable for empirical treatment of UTIs. Similar studies in Ghana and India have also reported very high levels of resistance to ampicillin and co-trimoxazole [20, 31]. The international clinical practice guidelines suggests that, trimethoprim/sulfamethoxazole should not be used empirically for the treatment of uncomplicated UTI in women if the level of resistance exceeds 20% [32].

Amongst the isolated uropathogens, 87.0% were found to be MDR, similar to a study in Ethiopia [30]. However, this level of MDR is much higher compared to what was reported in a study from Turkey, 53.8% [4]. Mostly, in low resource health facilities, clinicians do not have ready access to culture and sensitivity test results to guide their choice of medication. Therefore, suspected infections are empirically treated with a variety of antibiotics. This practice has the tendency to contribute to MDR. Most of the respondents reported they self-medicate, suggesting unregulated use of these antimicrobial agents. Furthermore, in the study area, there is little or no policy implementation on antibiotic sales, resulting in numerous unregulated antibiotics sales outlets. These factors could contribute to the observed high level of antimicrobial resistance.

This study also confirms ESBL presence within the *E. coli* and *Klebsiella* species warranting further surveillance. Given the common antimicrobial empiric management of UTI in The Gambia, due to scarce microbiological capabilities. Therefore, knowledge about the existing resistance patterns is paramount for antimicrobial stewardship.

Our findings show that having no formal education increased the risk of having uropathogenic bacterial infection. These findings are in contrast with a study reported from Ethiopia [6]. Formal education can significantly influence a person’s knowledge and therefore health seeking practices when it comes to exposure to infectious pathogens. Our study also showed that participants who had a previous history of UTI were twice as likely to have culture-positive results as those without a previous history of UTI. This result was consistent with a cross-sectional study conducted in Ethiopia [28] which found that history of UTI was a risk factor for uropathogenic infection. The possible explanation of this association could be due to the presence of resistant strains from earlier infections. Again, persons with previous UTI may still be exposed to the risk for recurrence.

The purchase and use of antibiotics without medical prescription was also found to be significantly associated with the presence of resistant uropathogenic bacterial isolates. This could be because of the presence of numerous drug outlets and lack of control of sales of antibiotics by over-the-counter medicine sellers. This practice could contribute to the development of antimicrobial resistant strains as reported in earlier works [17, 33–35]. This calls for antibiotic stewardship campaigns within the community to limit the use of unprescribed antibiotics. Similar to earlier reports from Uganda [9] and Sri-Lanka [36] we did not find an association between antibiotic use during the previous three months and the presence of resistant uropathogenic isolates. Also, the history of urethral catheterization was not found to be associated with the presence of uropathogenic bacteria [30].

## Conclusion

The current study found uropathogenic bacteria in patients with community-acquired UTI receiving care at a tertiary hospital in The Gambia. *Escherichia coli* was the predominant bacteria species identified. Having no formal education, history of UTI and the use of unprescribed antibiotics were found to be significant risks for current UTI. Nitrofurantoin, cefotaxime, and imipenem were observed to be the most sensitive antibiotics against the bacterial isolates. The high prevalence of MDR among the uropathogens isolated highlights the need for continuous AMR surveillance to update clinical protocol of UTI management.

Although some bacterial isolates were found to be multidrug resistant, molecular analysis were not done to identify the resistant genes. Most of the study participants were female which could have skewed the data, possible as a result of routine screening of pregnant women during antenatal care. These limitations notwithstanding, the current study provides valuable data on antibiotic susceptibility patterns of uropathogens in the study community to guide case management of UTI.

## Data Availability

The datasets used and/or analyzed during the current study are available from the corresponding author on reasonable request.

## Abbreviations

ATCC: American Type Culture Collection
AMR: Antimicrobial Resistance
CA-UTI: Community acquired Urinary Tract infection
CFU: Colony forming unit
CLSI: Clinical Laboratory and Standard Institute
KGH: Kanifing General Hospital
MDR: Multi-Drug Resistant
OPD: Outpatient Department
UTI: Urinary Tract Infection
WHO: World Health Organization
